# TPPU inhibits inflammation-induced excessive autophagy to restore the osteogenic differentiation potential of stem cells and improves alveolar ridge preservation

**DOI:** 10.1038/s41598-023-28710-0

**Published:** 2023-01-28

**Authors:** Haixia Dang, Weixian Chen, Lan Chen, Xinru Huo, Fu Wang

**Affiliations:** 1 The Affiliated Stomatological Hospital, Southwest Medical University, Luzhou, 646000 China; 2 School of Stomatology, Dalian Medical University, No. 9 West Section, Lvshun South Road, Dalian, 116044 China; 3 Academician Laboratory of Immune and Oral Development and Regeneration, Dalian Medical University, Dalian, 116044 China; 4 The Affiliated Stomatological Hospital of Dalian Medical University School of Stomatology, Dalian, 116086 China

**Keywords:** Adult stem cells, Mesenchymal stem cells, Stem-cell differentiation, Stem-cell research

## Abstract

Inflammation-induced autophagy is a double-edged sword. Dysfunction of autophagy impairs the differentiation capacity of mesenchymal stem cells and enhances inflammation-induced bone loss. Tooth extraction with periodontal and/or endodontic lesions exacerbates horizontal and vertical resorption of alveolar bone during the healing period. Alveolar socket preservation (ASP) procedure following tooth extraction has important clinical implications for future prosthodontic treatments. Studies have shown that epoxyeicosatrienoic acids (EETs) have significant anti-inflammatory effects and participate in autophagy. However, whether EETs can minimize alveolar bone resorption and contribute to ASP by regulating autophagy levels under inflammatory conditions remain elusive. Here, we figured out that LPS-induced inflammatory conditions increased the inflammatory cytokine and inhibited osteogenic differentiation of human dental pulp stem cells (hDPSCs), and led to excessive autophagy of hDPSCs. Moreover, we identified that increased EETs levels using TPPU, a soluble epoxide hydrolase inhibitor, reversed these negative outcomes. We further demonstrated the potential of TPPU to promote early healing of extraction sockets and ASP, and speculated that it was related to autophagy. Taken together, these results suggest that targeting inhibition of soluble epoxide hydrolase using TPPU plays a protective role in the differentiation and autophagy of mesenchymal stem cells and provides potential feasibility for applying TPPU for ASP, especially under inflammatory conditions.

## Introduction

Clinically, the most frequently extracted teeth are those with periodontal and/or endodontic lesions that result in bone destruction and aggravate alveolar bone resorption. Tooth extraction typically triggers a series of biological events which alter periodontal tissue homeostasis and structural integrity mediated by local inflammatory responses and periodontal tissue masticatory stimulation^1^. The alveolar ridge suffers horizontal and vertical resorption during the healing period following tooth extraction. Good preservation of the extraction socket site after extraction will help to achieve predictable and satisfactory results, especially in the anterior cosmetic area, which is of critical concern for implant placement because insufficient bone conditions may compromise the feasibility of implant and increase the likelihood of implant failure^2,3^.

Therefore, alveolar socket preservation (ASP), as an immediately guided bone regeneration procedure following tooth extraction, has important clinical implications for future prosthodontic treatments, including fixed bridges, removable partial dentures, complete dentures and implants restorations, by preventing or minimizing alveolar ridge resorption. Many different methods and materials have been reported for ASP, including autologous, allogeneic, xenograft and allogeneic bone grafts, and other materials such as bone morphogenetic protein (BMP), rich platelet plasma, platelet-rich fibrin (PRF), titanium-prepared platelet-rich fibrin (T-PRF) and Emdogain^3^. In the future, it is an important direction to seek a more effective and simple alveolar socket preservation strategy with multiple biological effects to attenuate the unfavorable factors affecting the healing of the extraction socket.

Infection and bone resorption following tooth extraction affect future restorative treatments^4^. Infection is one of the important reasons for bone resorption and delayed wound healing^5^.The extraction site is more prone to bacterial infection due to the unique environment of the oral cavity and oral hygiene. An infected socket induces an unstable and incomplete bone healing pattern. Several preclinical and clinical studies have shown that strong resorption of the alveolar bone and partial invagination of the mucosa often occur in the first few weeks after tooth extraction^6^. Therefore, maintaining the alveolar ridge condition and accelerating the healing of the extraction socket following tooth extraction are critical.

Autophagy can occur under physiological or pathological conditions and is a survival protection mechanism for maintaining cell physiological activities and homeostasis by degrading their own components. The autophagy process relies on various signaling mechanisms to achieve material recycling, and the changes in autophagy levels are related to stimuli such as starvation, infection, and oxidative stress^7^. Accumulating evidence suggests that autophagy is closely related to osteoclast, osteoblast, and osteocyte-mediated bone remodeling and plays a crucial role in coupling bone formation and bone resorption to maintain a normal postnatal bone steady state^8^. Some studies have shown that autophagy stimulates the primary cytoprotective mechanism of mesenchymal stem cells (MSCs) against stress, and autophagy dysfunction will impair the function of MSCs, leading to imbalanced bone remodeling and widespread aging and degenerative bone disease^9^. Studies have shown that autophagy is closely related to bone tissue regeneration, but the effect of autophagy on the preservation of dental sockets remains unclear. In addition to the critical role of autophagy in bone homeostasis, there is increasing evidence showing that autophagy is activated under different conditions, such as cellular starvation, inflammation, and oxidative stress. Autophagy also plays a crucial role in the resistance of many organisms to bacterial infection^10^. In the early stages of inflammation, autophagy acts as a protective mechanism, whereas in the later stages, excessive accumulation of autophagosomes may lead to tissue damage^11^. In the context of chronic inflammatory diseases, prolonged inflammation further complicates the regulation of autophagy. In recent years, chronic inflammatory diseases, including periodontitis, chronic arthritis and rheumatoid arthritis, have been associated with high levels of autophagy and bone loss, but the underlying mechanisms remain unclear^12^.

Dental stem cells provide new possibilities for regeneration with self-renewal, high proliferation and multi-directional differentiation potential. However, its biological characteristics, such as cell proliferation, differentiation, apoptosis and autophagy levels, vary under inflammatory conditions^13^. Studies have confirmed that LPS induces autophagy by Akt/mTOR signaling in A549 cells^14^. Upon exposure to LPS, the phosphorylated subunit p65 plays a vital role by triggering the transcription of specific genes, including *TNF-α*, *IL-1β* and *IL-6*^15^. Studies have shown that autophagy is a double-edged sword. The protective or deleterious effects of autophagy activation depend on cell type and stimuli context. Some studies have shown that autophagy can drive osteogenic differentiation of human gingival mesenchymal stem cells (HGMSC)^16^, while others have shown that increased autophagy by LPS inhibits the osteogenic differentiation of stem cells from apical papilla (SCAP)^17^. Therefore it is essential to understand the precise role and mechanisms of autophagy under inflammatory situations. Further research is required to understand the role of autophagy in the initiation and progression and how autophagy is linked with the other extrinsic factors following tooth extraction.

Epoxyeicosatrienoic acids (EETs) are derived from arachidonic acid (AA) metabolized by CYP450 cyclooxygenase (CYP450), which then are rapidly hydrolyzed by soluble epoxide hydrolase (sEH) to generate the corresponding four isomers of dihydroxyeicosatrienoic acids (DHETs, including 5,6-DHET, 8,9-DHET, 11,12-DHET and 14,15-DHET) with lower physiological activity. Therefore, inhibiting the degradation of EETs by sEH inhibitors (sEHi) has become a main method for EETs treatment^18^. Studies have shown that EETs have broad protective effects, including anti-inflammatory effects, preventing apoptosis, improving microcirculation, inhibiting osteoclasts and promoting bone tissue regeneration^19–23^. Studies have demonstrated that EETs/sEHi attenuates vascular inflammation and remodeling by inhibiting endothelial cell activation and reducing crosstalk between inflammatory cells and blood vessels^24^. In addition, studies have found that EETs/sEHi is associated with autophagy in alleviating inflammation. In an alcoholic cardiomyopathy model, studies have found that EETs induce autophagy, reduce apoptosis, and alleviate ethanol-induced myocardial dysfunction by regulating the amp-activated protein kinase (AMPK)/mTOR signaling pathway^22^. The sEH inhibitor AUDA increases autophagy and reduces apoptosis by activating the Nrf2 signaling pathway, alleviating myocardial remodeling and dysfunction in DCM^25^. t-AUCB pharmacologically modulates sEH in db/db mice, and significantly alleviates diabetic kidney injury by inhibiting inflammation and autophagy, relieving mitochondrial dysfunction and endoplasmic reticulum stress^26^.

Given the powerful effects of EETs, we hypothesized that EETs could promote the healing of extraction sockets and contribute to alveolar ridge preservation by regulating autophagy levels under inflammatory conditions. Here, we characterized the osteogenic differentiation and autophagy of human dental pulp stem cells (hDPSCs) under LPS-induced inflammatory conditions and identified that increased EETs levels using TPPU (an inhibitor of sEH) inhibit LPS-induced excessive autophagy and rescued osteogenic differentiation of hDPSCs. We further demonstrated the potential of TPPU to promote early healing of extraction sockets and ASP, and speculated that it was related to autophagy. Our results provide a potential possibility for the application of TPPU for ASP.

## Results

### TPPU protects hDPSCs against LPS-induced Inflammation and inhibition of osteogenic differentiation

**Figure 1:**
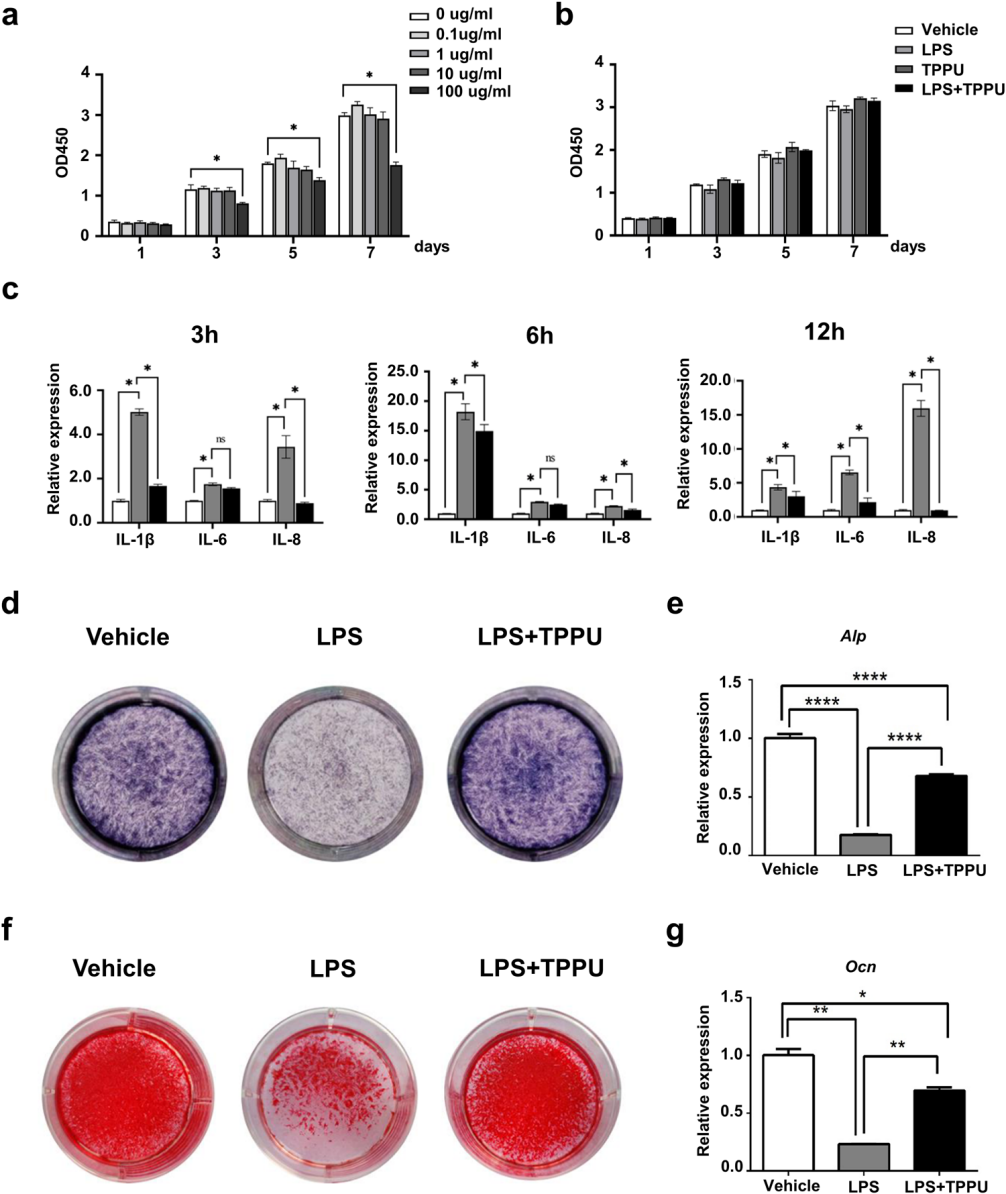
The effects of TPPU on the hDPSCs under LPS-induced inflammation conditions. (**a**) hDPSCs proliferation viability treated with different concentrations of LPS was tested by a CCK8 kit. (**b**) hDPSCs viability with/without TPPU treatment was measured by CCK8 under 10 μg/ml LPS-induced inflammatory conditions. (**c**) Relative mRNA expression levels of inflammatory factors *IL-1β, IL-6,* and *IL-8* in hDPSCs under LPS-induced inflammatory conditions in vitro with/without TPPU treatment for 3, 6 and 12 h, respectively. (**d**) ALP staining of hDPSCs, hDPSCs treated with LPS and hDPSCs treated with LPS and TPPU after 7 d of osteogenic induction. (**e**) *Alp* mRNA expression was evaluated by RT-qPCR in hDPSCs, hDPSCs treated with LPS and hDPSCs treated with LPS and TPPU after 7 d of osteogenic induction. (**f**) Alizarin red staining of hDPSCs, hDPSCs treated with LPS and hDPSCs treated with LPS and TPPU after 21 d of osteogenic induction. (**g**) *Ocn* mRNA expression in hDPSCs, hDPSCs treated with LPS and hDPSCs treated with LPS and TPPU was evaluated by RT-qPCR after 21 d of osteogenic induction. *P < 0.05; **P < 0.01; ***P < 0.001; ****P < 0.0001.

The cultured hDPSCs were positive for CD44, CD73, CD90, CD105, and STRO-1, and negative for CD34 and CD45 (Supplementary Fig. 1). To exclude the detrimental effects of lethal toxicity of inappropriate LPS concentration on hDPSCs viability, we treated hDPSCs with various concentrations of LPS for 7 days. The CCK8 assay showed that 100 μg/ml LPS remarkably decreased the cell viability on days 3, 5 and 7 of culture; while 0.1 μg/ml, 1 μg/ml and 10 μg/ml LPS had no significant difference in cell viability (Fig. 1a). Thus, combining our data and referring to other studies, we used 10 μg/ml of LPS for subsequent experiments. Next, we detected the effect of TPPU on the proliferation of hDPSCs (Fig. 1b). The results showed that TPPU had no significant effect on cell viability of hDPSCs with or without LPS. Then we detected the anti-inflammatory effect of TPPU. RT-qPCR analysis demonstrated that the expression of inflammatory factors *IL-1β*, *IL-6* and *IL-8* was significantly increased in hDPSCs treated with LPS for 3 h, 6 h and 12 h, respectively, while TPPU significantly inhibited the LPS-induced expression of *IL-1β, IL-6* and *IL-8* after 3 h, 6 h and 12 h (Fig. 1c). These results suggested that TPPU reduced inflammatory factors in hDPSCs induced by LPS.

The differentiation potential of hDPSCs may be changed under inflammatory conditions, which in turn affects tissue regeneration. Therefore, maintaining the differentiation ability of hDPSCs is the basis of tissue regeneration under inflammation. We then evaluated the effect of TPPU on the differentiation of hDPSCs under LPS-induced inflammation. Alkaline phosphatase (ALP) staining and alizarin red (ARS) staining demonstrated that 10 μg/ml of LPS weakened the osteogenic differentiation of hDPSCs, while TPPU significantly alleviated LPS-induced impairment of hDPSCs osteogenic differentiation (Fig. 1d,f) and increased the expression of osteogenesis-related genes *Alp* and *Ocn* (Fig. 1e,g). The results suggested that TPPU rescued the LPS-induced osteogenesis inhibition.

### TPPU attenuates LPS-induced excessive autophagy

**Figure 2:**
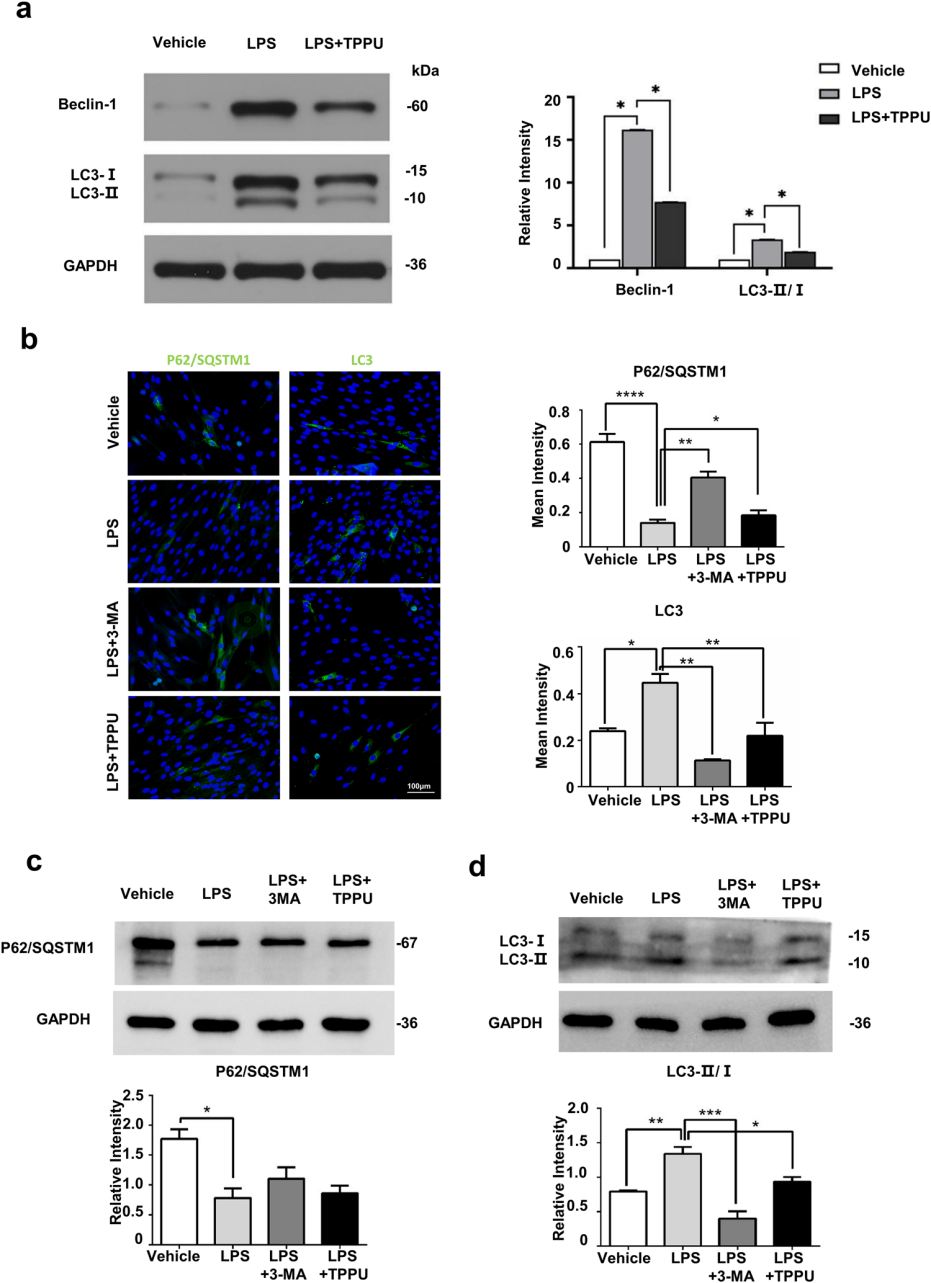
TPPU inhibiting LPS-induced abnormal autophagy. (**a**) The protein expression of Beclin-1, LC3 and relative quantification in hDPSCs, hDPSCs treated with LPS and hDPSCs treated with LPS and TPPU following 24 h of osteogenic induction. Uncropped Western blot images are shown in Supplementary Fig. 4a. (**b**) Immunofluorescence and quantitative analysis of P62/SQSTM1 and LC3 expression in hDPSCs, hDPSCs treated with LPS and hDPSCs treated with LPS and TPPU following 24 h of osteogenic induction. (**c**, **d**) Western blotting for the P62/SQSTM1 and LC3 expression in hDPSCs, hDPSCs treated with LPS, hDPSCs treated with LPS and 3-MA, hDPSCs treated with LPS and TPPU following 24 h of osteogenic induction. Uncropped Western blot images are shown in Supplementary Fig. 4b, c. *P < 0.05; **P < 0.01; ***P < 0.001; ****P < 0.0001.

Studies have reported that inflammatory cytokines increase the level of autophagy in cells, and excessive autophagy induces apoptosis and cell damage, while sEHi can alleviate inflammation-induced autophagy. We therefore examined the effect of TPPU on the autophagy level in osteogenic differentiation of hDPSCs under inflammatory conditions. Western blot assay showed that LPS significantly increased autophagy-related proteins LC3II/I and Beclin-1, while TPPU substantially reduced the LC3II/I and Beclin-1 protein level (Fig. 2a). To further explore whether autophagy was involved in alleviating inflammation by TPPU, we treated the hDPSCs with the autophagy inhibitor 3-MA under inflammatory conditions for 24 h or 7 days. We detected the expression of P62/SQSTM1 (a ubiquitin-binding protein involved in cell signaling, oxidative stress, and autophagy) and LC3. Immunofluorescence assay confirmed that the fluorescence intensity of P62/SQSTM1 or LC3 in the LPS group was significantly lower or higher than that in the control group. TPPU treatment played the same role as 3-MA which rescued excessive autophagy flux of hDPSCs under inflammatory conditions with the increased p62 fluorescence intensity and decreased the number of punctate LC3 dots (Fig. 2b). Western blotting assay showed that the expression of P62/SQSTM1 and LC3 was similar but not exactly the same expression trend to immunofluorescence assay (Fig. 2c,d). To investigate the effect of LPS and TPPU on autophagy at different time points after 3-MA treatment, we also added the P62 protein expression assay after 48 h of drug treatment, which was consistent with the P62 expression trend after 24 h of drug treatment (Supplementary Fig. 2). Our results demonstrated that TPPU attenuated LPS-induced abnormal autophagy flux.

### TPPU promotes osteogenic differentiation of hDPSCs via regulating autophagy under inflammatory microenvironments

**Figure 3:**
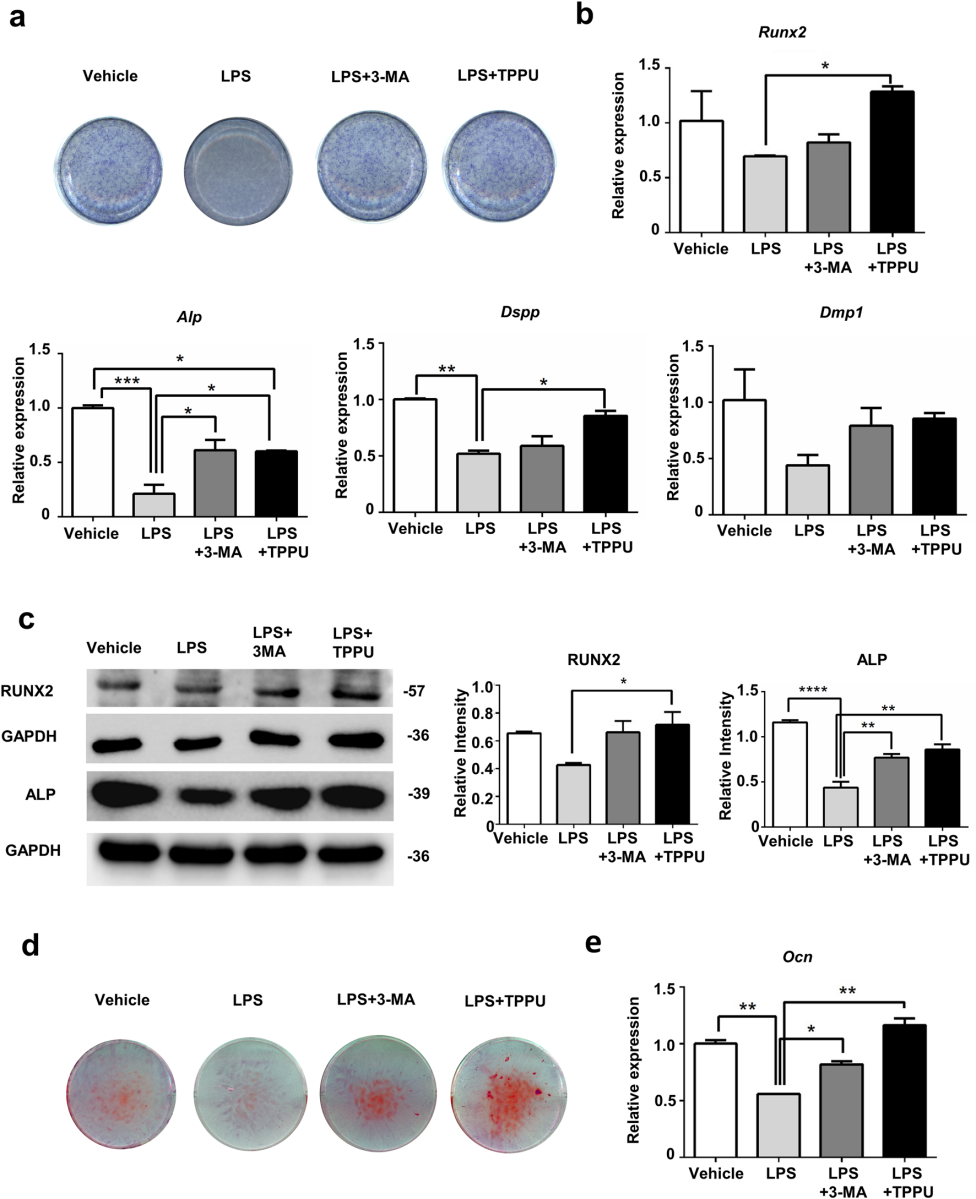
TPPU restoring the impaired osteogenic differentiation potential of hDPSCs under inflammatory conditions by inhibiting autophagy. hDPSCs were treated with LPS, treated with LPS and 3MA and treated with LPS and TPPU under osteogenic induction for 7 or 21 days. (**a**) ALP staining of hDPSCs after 7 d of osteogenic induction. (**b**) RT-qPCR assay of the expression of osteogenesis-related factors *Runx2*, *Alp, Dspp and Dmp1* in hDPSCs after 7 d of osteogenic induction. (**c**) Western blotting showing the protein expression and quantitative analysis of RUNX2 and ALP. Uncropped Western blot images are shown in Supplementary Fig. 5. (**d**) Alizarin red staining of hDPSCs after 21 d of osteogenic induction. (**e**) RT-qPCR assay of the expression of osteogenesis-related factors *Ocn* in hDPSCs after 21 d of osteogenic induction. *P < 0.05; **P < 0.01; ***P < 0.001; ****P < 0.0001.

We next explored whether TPPU promoting the osteogenic differentiation of hDPSCs under an inflammatory microenvironment was related to its mitigation of inflammation-induced excessive autophagy. ALP staining of hDPSCs after seven days of culture showed that the osteogenic impairment induced by the inflammatory environment could be rescued by the addition of TPPU, and a similar effect could be achieved by the addition of 3-MA (Fig. 3a). RT-qPCR confirmed that TPPU and 3-MA had the same effect on increasing the expression of *Runx2, Alp, Dspp and Dmp1* in hDPSCs after7 days of osteogenic induction (Fig. 3b). Our previous evidence suggested that TPPU significantly modulates LPS-induced autophagy in the early stages. We therefore examined osteogenesis-related protein levels in hDPSCs 7 days after osteogenic induction under inflammatory conditions. We found that TPPU and 3-MA also had the same effect on significantly increasing the protein level of RUNX2 and ALP under the inflammatory environment (Fig. 3c). The trend of AR staining and *Ocn* mRNA expression in hDPSCs cultured for 21 days of osteogenic induction was consistent in hDPSCs after 7 days of osteogenic induction (Fig. 3d,e). In addition, we also treated the cells by combining the autophagy promoters rapamycin and LPS, rapamycin and TPPU, cultured them in osteogenic induction medium for 7 days, and detected the changes in their osteogenic status by RT-qPCR (Supplementary Fig. 3). The results showed that rapamycin exacerbated poor osteogenesis due to autophagy in an inflammatory environment, while TPPU was able to rescue this trend to some extent. These findings indicated that TPPU restoring the osteogenic differentiation potential of hDPSCs under an inflammatory microenvironment was associated with reducing inflammation-induced excessive autophagy.

### TPPU improves extraction socket healing and ASP

**Figure 4:**
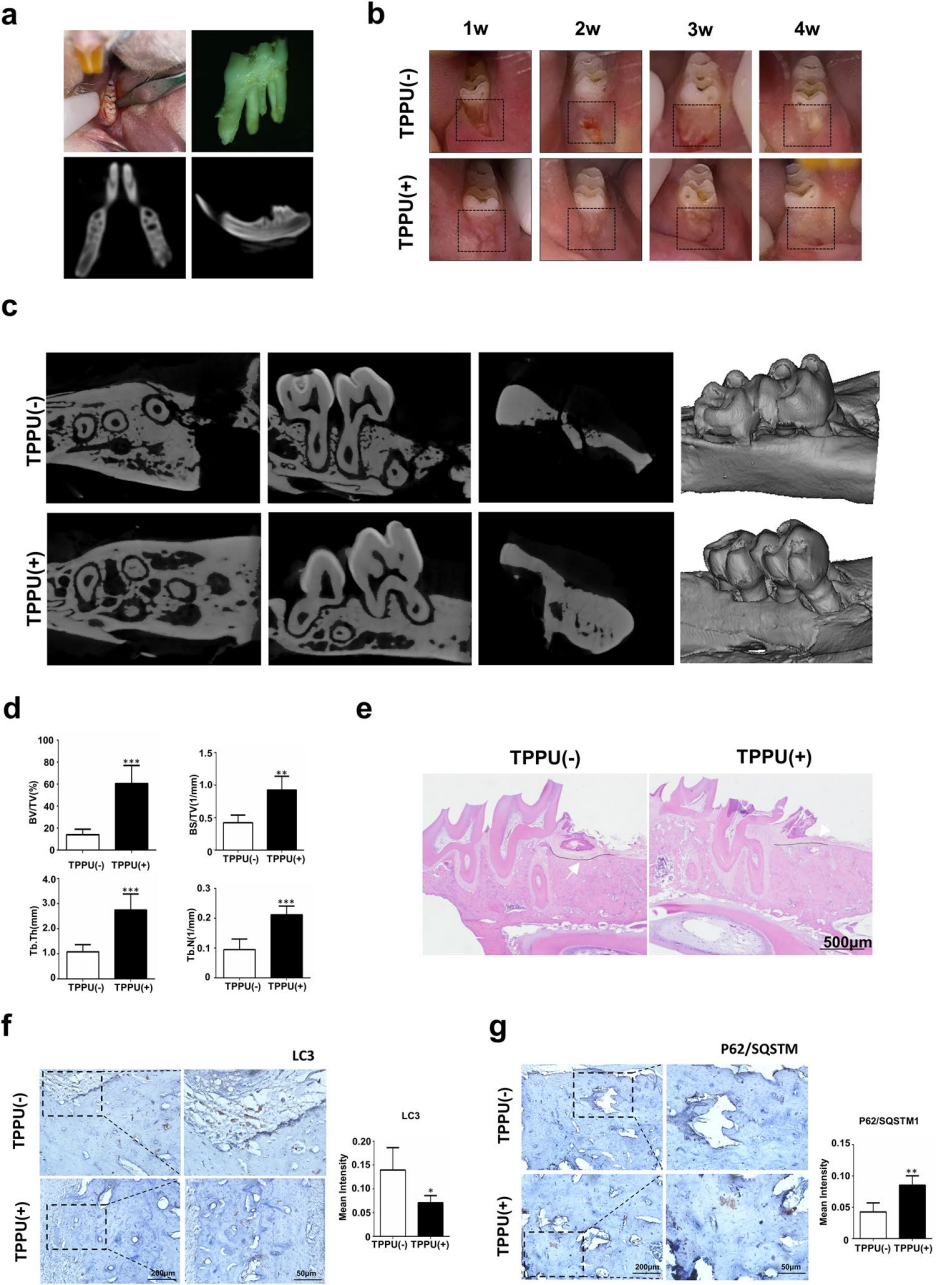
TPPU reduces alveolar ridge resorption after tooth extraction. (**a**) Tooth extraction procedure and MicroCT examination of extraction socket. (**b**) Representative images of the healing of extraction sockets treated with/without TPPU from 1 week through 4 weeks after tooth extraction. (**c**, **d**) Representative MicroCT images of the mandible 4 weeks after tooth extraction in TPPU treatment and control groups, and quantifications of bone parameters of extraction socket region (n = 4). BV/TV, the percentage bone volume; BS/TV, bone surface density; Tb.Th, trabecular thickness; Tb.N, trabecular number. Recon (v.7.4.2) was used for reconstruction and 3D mages were contoured from 2D (CTvox, version 3.3.0). 3D and 2D images were analyzed by CTan (version 1.18.8.0, https://blue-scientific.com/bruker-ctan-micro-ct-software). (**e**) Representative HE staining of jawbone. (**f**, **g**) Immunohistochemical detection of LC3 and P62 expression in tooth extraction socket area after 4 weeks of tooth extraction. *P < 0.05; **P < 0.01; ***P < 0.001; ****P < 0.0001.

To further explore whether TPPU reduced the early alveolar bone resorption following tooth extraction, we established a tooth extraction socket model in SD rats and administered topical TPPU (Fig. 4a). We observed that TPPU-treated extraction sockets healed faster and better with complete soft tissue closure 4 weeks after tooth extraction. In contrast, extraction sockets without TPPU treatment healed slightly slower, showing inflammatory features with somewhat red and swollen soft tissue, which did not completely close until 6 weeks (Fig. 4b). After 4 weeks of treatment, MicroCT assay of mandibular bone indicated that the average width in TPPU-treated socket was wider than that in control group, and extraction area treated with TPPU had significantly higher bone mineral density (BMD) than the control group (Fig. 4c). CTAn software analysis showed that BV/TV, BS/TV, Tb.Th and Tb.N were significantly increased in TPPU-treated sockets, indicating a decrease in bone remodeling (Fig. 4d).

HE staining showed that the soft tissue in TPPU treatment group healed well, and the level of the alveolar ridge was consistent with the cementoenamel boundary of the second molar. The tissue was characterized by the structure of mature bone with dense and evenly arranged trabecular bone and with almost no alveolar bone resorption (Fig. 4e). On the contrary, the amount of newly formed bones was severely reduced in control group compared to TPPU treatment group, and the height of the alveolar ridge in the extraction socket was significantly lower than the cementoenamel boundary of the second molar. We also examined the expression of LC3 and P62 in tooth extraction socket area after 4 weeks of tooth extraction using immunohistochemistry and found that LC3 expression was significantly decreased and P62 expression was significantly upregulated in the extraction socket in the TPPU-treatment group compared with control group (Fig. 4f,g), which was consistent with the in vitro results. These results suggested that TPPU reduced inflammation and autophagy of extraction sockets, contributing to ASP.

## Discussion

Wound healing of extraction sockets is a very complex process, including hemostasis, inflammation, proliferation and remodeling, which often leads to the vertical and horizontal resorption of alveolar ridges and negatively affects functional and aesthetic results. Bone remodeling of extraction sockets persists until 4–6 months after extraction with marked changes in the height and width of the alveolar ridge^3^. Based on the unique environment of the oral cavity, bone destruction resulting from periodontal or endodontic disease and post-extraction infection complications can aggravate alveolar bone resorption. Therefore, preventing an inappropriate inflammatory environment in the extraction socket and maintaining the height and width of alveolar ridge following tooth extraction is critical for functional restorations. Many methods have been used to reduce alveolar ridge resorption after tooth extraction, including bone grafting, bone substitutes, minimally invasive tooth extraction and collagen plugs. Our present study in vitro showed that TPPU reduced the inflammatory cytokines and reversed LPS-induced excessive autophagy to restore the osteogenic differentiation potential of hDPSCs. In vivo results demonstrated that TPPU applied to the fresh extraction sockets positively influenced socket healing, reduced alveolar ridge resorption following tooth extractions.

Some studies have found that stem cells are prone to aging in the inflammatory microenvironment, which affects the proliferation and differentiation ability of stem cells^27^. LPS exerts an inhibitory effect on the osteogenic and odontogenic differentiation of apical dental papilla stem cells^17^. It has been found that autophagy plays an important role in the self-renewal and differentiation of stem cells at both the cellular and molecular levels^8^. However, the effects of autophagy on the stemness maintenance, self-renewal and differentiation of stem cells, and the underlying molecular mechanisms still need to be further studied.

Autophagy is activated under different conditions, including cell starvation, inflammation, and oxidative stress. Autophagy affects cellular processes such as inflammation, apoptosis, redox balance, and proliferation^28,29^. In response to stress, autophagy acts as a protective mechanism. Studies have shown that activation of autophagy can protect dental pulp cells from LPS-induced inflammatory damage and CoCl2-induced hypoxic stress damage in hDPSCs^30,31^. In addition, the pharmacological effects of many drugs are also related to the enhancement of autophagy^32^. However, overactivation of autophagy may induce apoptosis and promote cell death^33^. Studies have found that autophagy has dual effects on the osteogenic differentiation of mesenchymal stem cells. Autophagy can drive osteogenic differentiation of human gingival mesenchymal stem cells, when inhibiting autophagy greatly reduced osteogenic marker expression^16^. Autophagy stimulation by rapamycin reverses the low osteogenic capability of bone marrow MSCs in osteoporotic patients. However, inflammation-induced activation of ‘excessive’ autophagy can affect the differentiation of MSCs, especially in the early stages of differentiation^7^. LPS can enhance autophagy and reduce the differentiation capacity of apical papilla stem cells, while autophagy inhibitors restore the expression of differentiation genes *Alp* and *Runx2*^17^.

EETs are involved in cell proliferation, cell migration, and angiogenesis. EETs are essential in maintaining vascular homeostasis, inhibiting vascular inflammation, and promoting angiogenesis. EETs/sEHi have the property of promoting the regeneration of different tissues^34^. EETs have been shown to have inflammatory protective effects in various types of arthritisand neuritis^35,36^. TPPU stabilizes the level of EETs and exerts an anti-inflammatory effect by reducing the expression of NLRP3 inflammasome-related molecules under LPS stimulation^37^ and inhibiting NF-kB activation in mouse macrophages^38^. Besides, Some studies have found that TPPU can affect the interaction between oxidative stress and inflammatory response. Some studies have found that TPPU can increase neuron survival and memory to a certain extent, reduce the occurrence of oxidative stress and inflammation, and found that EETs can improve neuroinflammation, neuronal death and oxidative stress by modulating gsk3β-mediated NF-κB, p53 and Nrf2 signaling pathways^39–41^. Another preclinical model study has demonstrated that TPPU treatment can significantly reduce the apoptosis and oxidative stress of transplanted stem cells, thereby significantly increasing the survival of transplanted stem cells^42^.

Studies have reported that EETs are involved in autophagy. 14,15-EET has an inflammatory protective effect on human bronchial epithelial cells induced by cigarette smoke by inhibiting autophagy and promoting the expression of anti-inflammatory cytokines^43^. Autophagy is inhibited in Ephx2 deficiency mouse model^44^. EETs protect the heart and liver by autophagy regulation^45,46^. Our previous study has shown that TPPU can significantly increase angiogenesis, and reduce acinar cell apoptosis and histological damage in ir-injured mice^47^.

Taken together, the extraction socket undergoes a series of complex changes after tooth extraction and is susceptible to inflammation due to the special environment of the oral cavity. Reducing the adverse effects of inflammation for well-healed alveolar socket after tooth extraction using appropriate ASP techniques is essential for future restorative treatments. TPPU can promote the healing of tooth extraction socket and contribute to ASP by regulating inflammation-induced excessive autophagy. Future developing materials with controlled release for TPPU administration for ASP are worthy of further research.

## Conclusions

Our results confirm that TPPU restored impaired osteogenic differentiation potential of hDPSCs caused by inflammation-induced excessive autophagy. In vivo experiments further show the promise of TPPU for ASP by inhibiting inflammation and might be related to the autophagy level. Our results provide potential feasibility for applying TPPU for ASP by regulating autophagy, especially in infection situations.

## Methods

### Chemicals

TPPU (Sigma‑Aldrich, Merck KGaA, Darmstadt, Germany) was dissolved in dimethyl sulfoxide (DMSO, Coolaber, Beijing, China) as a stock solution (10 mg/ml), and further diluted to 10 μM TPPU for cell treatment in vitro or 20 μM TPPU for local injection *in vivo*^26,47^. Cells were treated with or without an autophagy inhibitor 3-methyladenine (3-MA, 10 mM; Solarbio, Beijing, China), an autophagy inducer Rapamycin (Rapa, 5 µM; Solarbio, Beijing, China).

### Animal and tooth extraction model

SD rats were housed under standard specific-pathogen-free conditions and all animal experiments were performed in accordance with protocols that were approved and authorized by the Ethics Committee of Dalian Medical University (AEE-22004) and conformed to Institutional Animal Care and Use Committee (IACUC) guidelines and regulations of Dalian Medical University for laboratory animal use. All animal experiments were carried out in compliance with the ARRIVE guidelines.

Twenty male SD rats (body weight 110–120 g, approximately 8 weeks old) were obtained from the Experimental Animal Center of Dalian Medical University. The mandibular first molars were extracted bilaterally to establish an extraction socket model under anesthesia, the absorbable gelatin sponge was then placed in the extraction socket. Considering the possible influence of saliva and tooth extraction socket bleeding in the oral cavity of rats on TPPU concentration, we adopted a 2 × TPPU for cytological dose. The next day, 10 μl of TPPU (20 μM) or PBS was injected into extraction area once a day for 7 days. All animals were euthanized 4 weeks after tooth extractions. Jaws including the extraction site were resected for Micro-CT and immunohistochemical staining.

### Isolation and culture of human dental pulp stem cells and treatment

This study was approved by the Ethics Committee of the Affiliated Stomatological Hospital of Dalian Medical University School of Stomatology per the Declaration of Helsinki (No. 2022001). Premolar teeth were collected from children (aged 14–19 years) undergoing orthodontic treatment and informed consents were obtained from all donors in accordance with relevant guidelines and regulations. Human dental pulp stem cells (hDPSCs) were isolated from extracted orthodontic teeth following the established method^44^, then were cultured and expanded in Dulbecco's Modified Eagle Medium/Nutrient Mixture F-12 (DMEM/F12, Hyclone, Logan, UT, USA) supplemented with 10% fetal bovine serum (FBS, Gibco, Thermo Fisher, MA, USA) and 1% penicillin-streptomycin (Gibco, Thermo Fisher, Inc, MA, USA) and at 37 °C in 5% CO2. Cell passage 5 was used for experiments. The cultured cells were performed Flow cytometry staining with CD34-APC, CD44-PE, CD45-FITC, CD73-PE, CD90-FITC, CD105-FITC, STRO-1-PE antibodies (BD Biosciences, East Rutherford, USA), and then samples were analyzed using a FACSVerse flow cytometer (BD, USA). To pharmacologically inhibit sEH, cells were treated with or without 10 μM TPPU according to our previous study^47^.

### Cell viability assay

hDPSCs were seeded in a 96-well plates (8000 cells/well). Different concentrations of reagents were administered to the experimental groups. After culturing for 1, 3, 5, and 7 days, 10 μL of CCK8 (ApexBio Technology, Texas, USA) reagent was added to each well to continue incubating for 1 h at 37 C, and the absorbance at 450 nm was measured with a microplate reader.

### Quantitative real-time polymerase chain reaction (RT-qPCR)

Total RNA was extracted from hDPSCs with RNAiso Plus (TRIzol; Takara Bio, Otsu, Japan), and was reverse transcribed to cDNA using HiScript II Q RT Super Mix (Vazyme Biotech, Nanjing, China) according to the manufacturer’s protocol. The primers used for RT-qPCR were as follows: GAPDH (forward, 5’-GTGAAGGTCGGAGTCAACG-3’; reverse, 3’-TGAGGTCAATGAAGGGG TC-5’), IL-1β (forward, 5’-TGGCTTATTACAGTGGCAATGAGGAT-3’; reverse, 3’-TGTAGTGGTGGTCGGAGATTCGTAG-5’), IL-6 (forward, 5’-GTTGGGTCAGGGGTGGTTATTGC-3’; reverse, 3’-TGAGAGTAGTGAGGAACAAGCCAGAG-5’), IL-8 (forward, 5’- CTCTCTTGGCAGCCTTCCTGATTTC-3’; reverse, 3’-TTTGGGGTGGAAAGGTTTGGAGTATG-5’), RUNX2 (forward, 5’-AGCAAGGTTCAACGATCTGAGAT-3’ ; reverse, 3’-TTTGTGAAGACGGTTATGGTCAA-5’), OCN (forward, 5’-CTACCTGTATCAATGGCTGG-3’; reverse, 3’-GGATTGAGCTCACACACCT-5’). GAPDH served as the internal control. RT-qPCR was performed using ChamQ Universal SYBR qPCR Master Mix. The relative expressions of genes were calculated by the 2^−ΔΔCT^ method.

### Osteogenic differentiation and assay

hDPSCs were seeded in 6-well plates (3 × 10^5^ cells/well) and were cultured in osteogenic medium (DMEM supplemented with 10% FBS, 100 nmol/L dexamethasone, 10 mmol/L β-glycerol phosphate, and 50 μmol/L ascorbate-2-phosphate) after the cells reached 80% confluence. ALP staining, ARS staining and semi-quantitative analysis were performed as previously described^48^.

### Western blotting

As previously mentioned^48^, proteins were isolated and electroblotted. The membranes were blocked for 1 h with 5% BSA (ZSGB-BIO, Beijing, China) and incubated overnight at 4℃ with the primary antibodies (anti-Beclin-1, 1:1000dilution; anti-LC3, 1:2000 dilution; anti-RUNX2, 1:1000 dilution; anti-P62/SQSTM1, 1:5000 dilution, all from Abcam, MA, USA). After washing, membranes were incubated for 1 h with the secondary horseradish peroxidase antibody (Solarbio, Beijing, China). Finally, the intensity of the bands was determined using the Bio-Rad VersaDoc imaging system and the ECL kit (Solarbio, Beijing, China).

### Immunofluorescence and immunohistochemical staining

For immunofluorescence staining, the cells seeded on collagen-coated glass slides were fixed in 4% formaldehyde and treated with phosphate-buffered saline (PBS) with 0.5% Triton X-100 for permeabilization. The sections were incubated with the primary antibodies anti-P62/SQSTM1 (1:1000 dilution, Abcam, MA, USA) overnight at 4 °C, After incubation with primary antibodies, sections were washed with PBS three times and were incubated with secondary antibodies conjugated with Cy3 568 and dylight 488 (1:200 dilution, Abcam, MA, USA) for 1 h at 37 °C. Sections were then counterstained with 4’,6-diamidino-2-phenylindole (DAPI). Cells were visualized using fluorescence microscopy.

For immunohistochemical staining, jaws were fixed and decalcified, embedded in paraffin. The embedded tissues were sectioned at 6 μm, deparaffinized, and subjected to hematoxylin and eosin (H&E) staining and Masson's trichrome staining (Solarbio, Beijing, China) according to the manufacturer’s instruction.

### Micro-CT imaging and analysis

The mouse jaw bones were isolated and preserved for 48 h in 4% paraformaldehyde. Then, the harvested jaw bones were scanned with a microCT scanning system (Skyscan 1276, Bruker Micro-CT, Belgium) at 6.5 μm voxels, 85 kV, 200 μA, 1 mm Al filter. The three-dimensional reconstruction using Recon (version1.7.4.2). A cylindrical volume of interest (ROI) centered around extraction site was selected with dimensions of 2 mm × 2 mm × 2 mm. The parameters including percentage bone volume (BV/TV), bone surface density (BS/TV), Trabecular thickness (Tb.Th) and Trabecular number (Tb.N) were analyzed using CTAn software (version.v.1.18).

### Statistical analysis

At least three times each of the tests were repeated. GraphPad Prism 9 (CA, USA) was employed for Student-Newman-Keuls tests and one-way ANOVA. Data were considered statistically significant at *p* < 0.05 and showed the means ± SEM of replicate measurements.

## Supplementary Information

Supplementary Information.

## Data Availability

Te data used to support the fndings of this study are available from the corresponding author upon request.
