# Supplementary material for: TPPU inhibits inflammation-induced excessive autophagy to restore the osteogenic differentiation potential of stem cells and improves alveolar ridge preservation
Source: Sci Rep. 2023 Jan 28;13:1574. doi: 10.1038/s41598-023-28710-0 (PMC9884285; doi:10.1038/s41598-023-28710-0)
Supplement: Supplementary file 1 — Supplementary Information. [file 41598_2023_28710_MOESM1_ESM.pdf]

Supplementary Information for:

TPPU inhibits inflammation-induced excessive autophagy to restore the osteogenic differentiation potential of stem cells and improves alveolar ridge preservation

Haixia Dang[1,2+], Weixian Chen[2,3+], Lan Chen[1], Xinru Huo[2], Fu Wang[2,3,4]*

[1]The Affiliated Stomatological Hospital, Southwest Medical University, Luzhou, 646000, China.
[2]School of Stomatology, Dalian Medical University, Dalian, 116044, China.
[3]Academician Laboratory of Immune and Oral Development & Regeneration, Dalian Medical University, Dalian, 116044, China.
[4]The Affiliated Stomatological Hospital of Dalian Medical University School of Stomatology, Dalian, 116086, China.

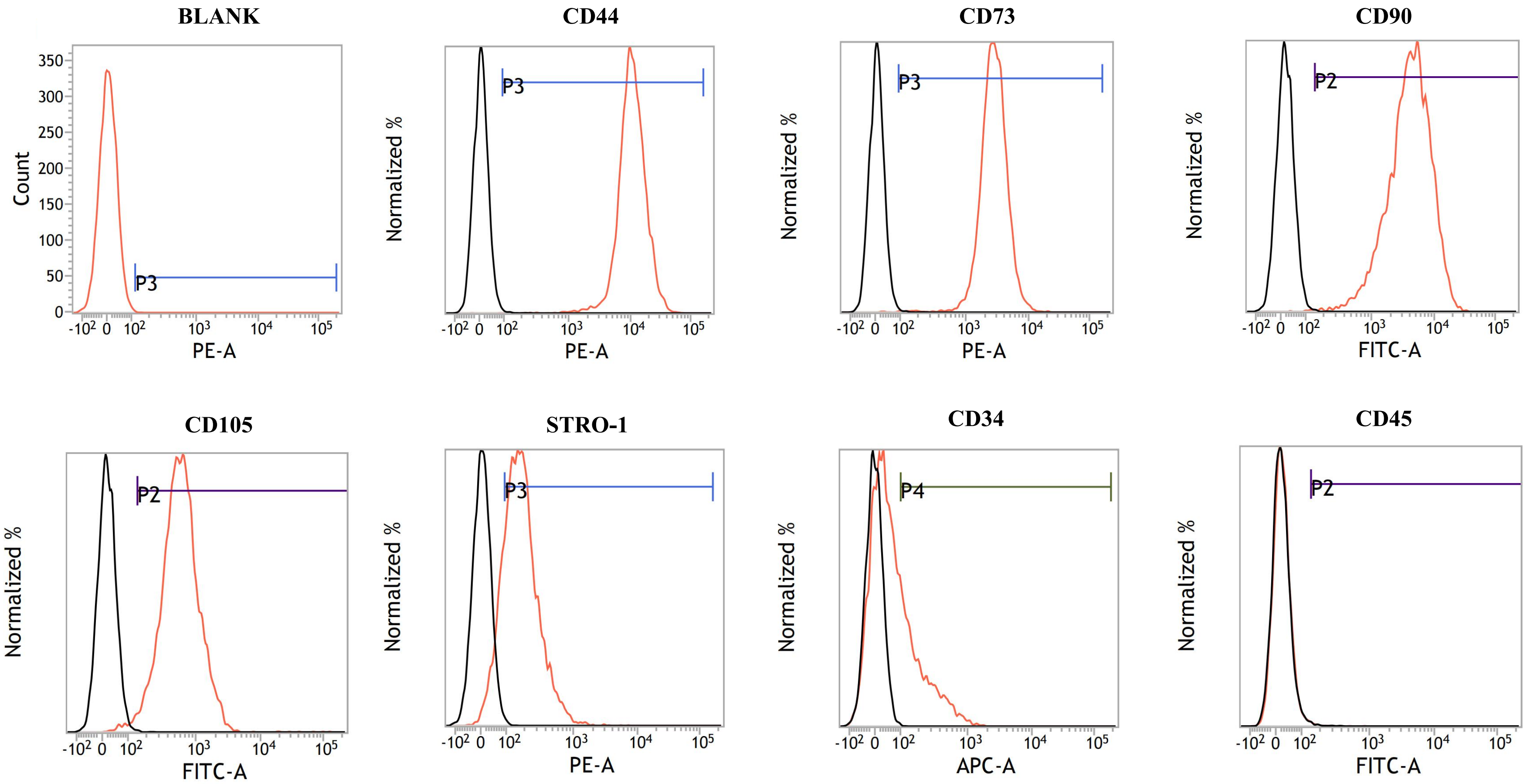

Supplementary Fig. 1 Flow cytometry showing stem cells surface makers. The hDPSCs are positive for CD44, CD73, CD90, CD105, and STRO-1, and negative for CD34 and CD45. The black histograms show the isotype control-stained cells and the red histograms show the antibody-stained cells. The control population was stained with matched isotype antibodies.

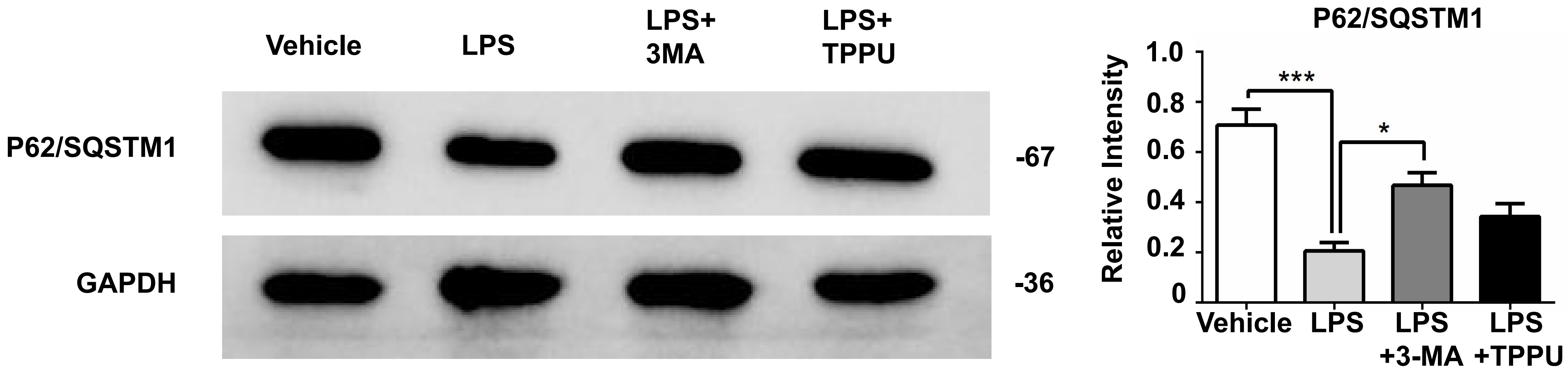

Supplementary Fig. 2 Western blotting for the P62/SQSTM1 expression in hDPSCs. hDPSCs treated with LPS, hDPSCs treated with LPS and 3MA, hDPSCs treated with LPS and TPPU following 48 h of osteogenic induction.Uncropped Western blot images are shown in Supplementary Fig. 6.

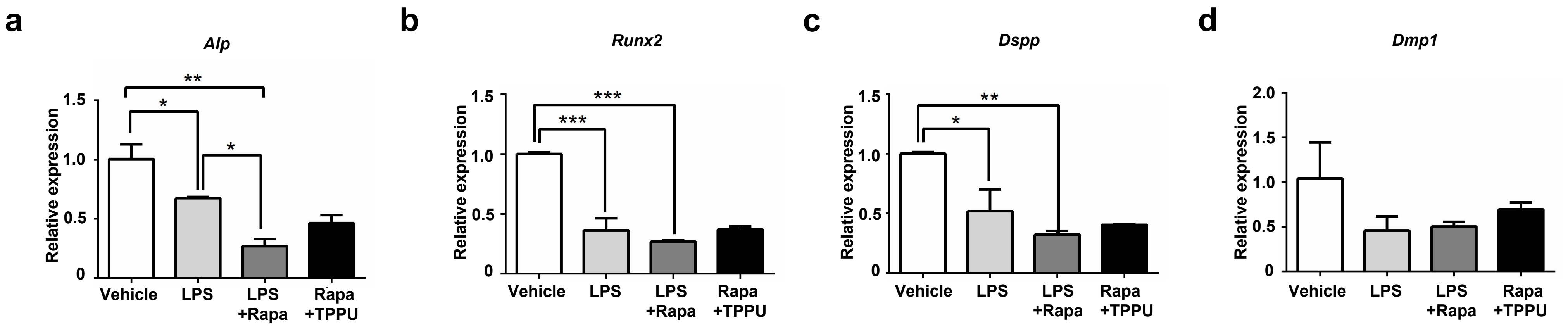

Supplementary Fig. 3 mRNA expression of *Alp, Runx2, Dspp, Dmp1* was evaluated by RT-qPCR in hDPSCs. hDPSCs treated with LPS, LPS and Rapa, Rapa and TPPU following 7 d of osteogenic induction.

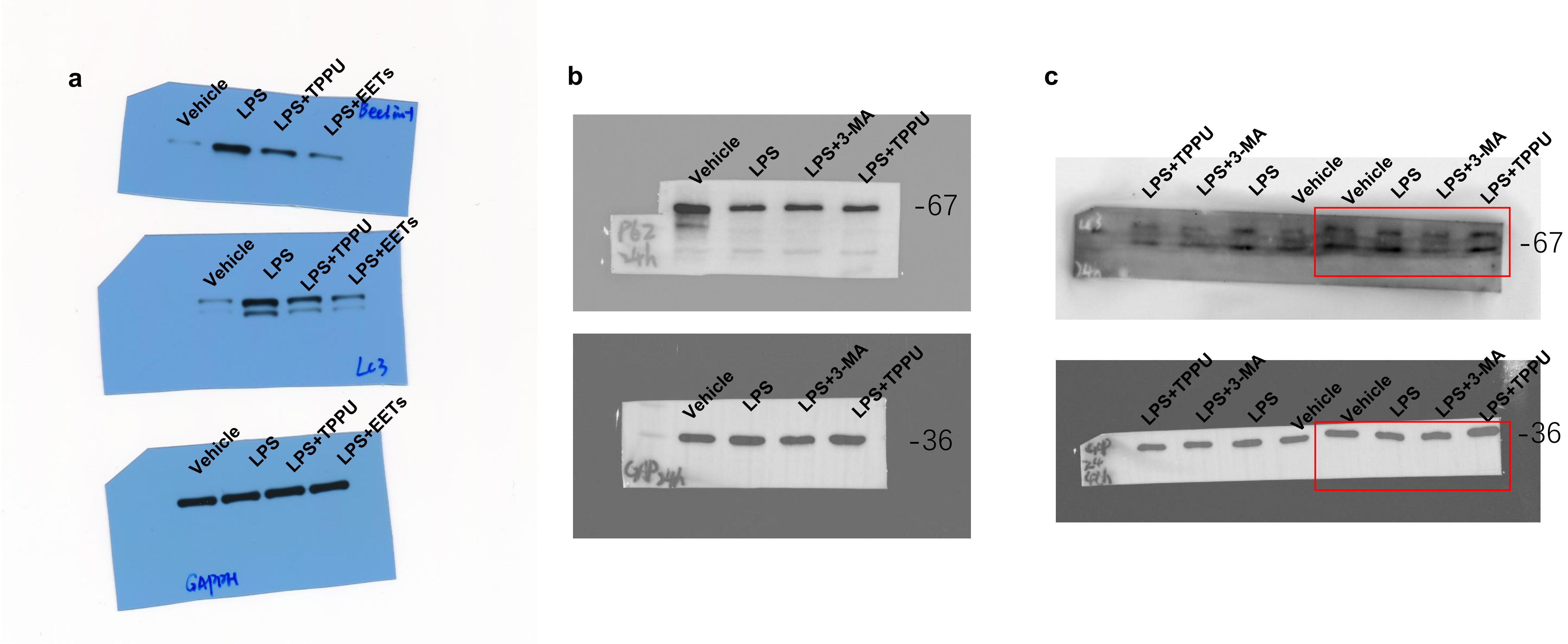

Supplementary Fig. 4 Unprocessed western blots and/or gels. Source Data for Figure2a (a); Source Data for Figure2c (b); Source Data for Figure2d (c)

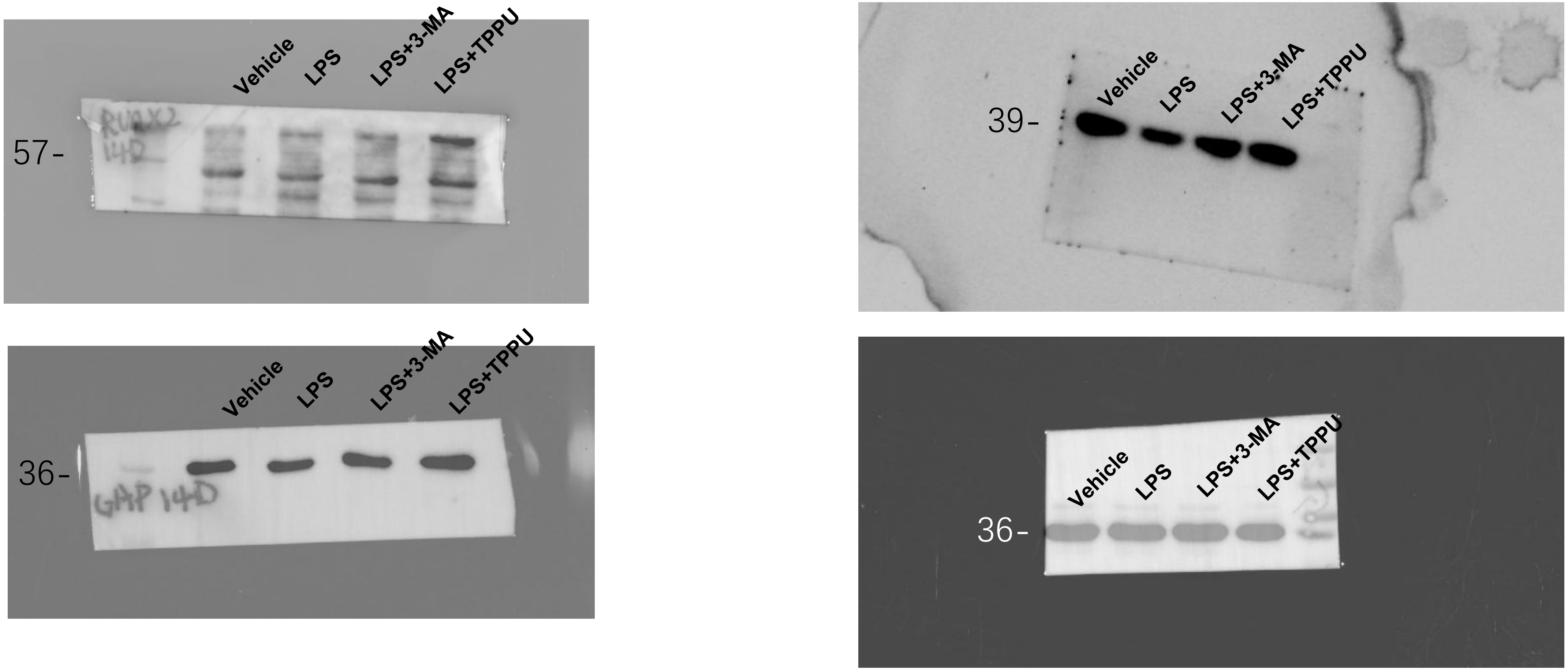

Supplementary Fig. 5 Unprocessed western blots and/or gels. Source Data for Figure 3c

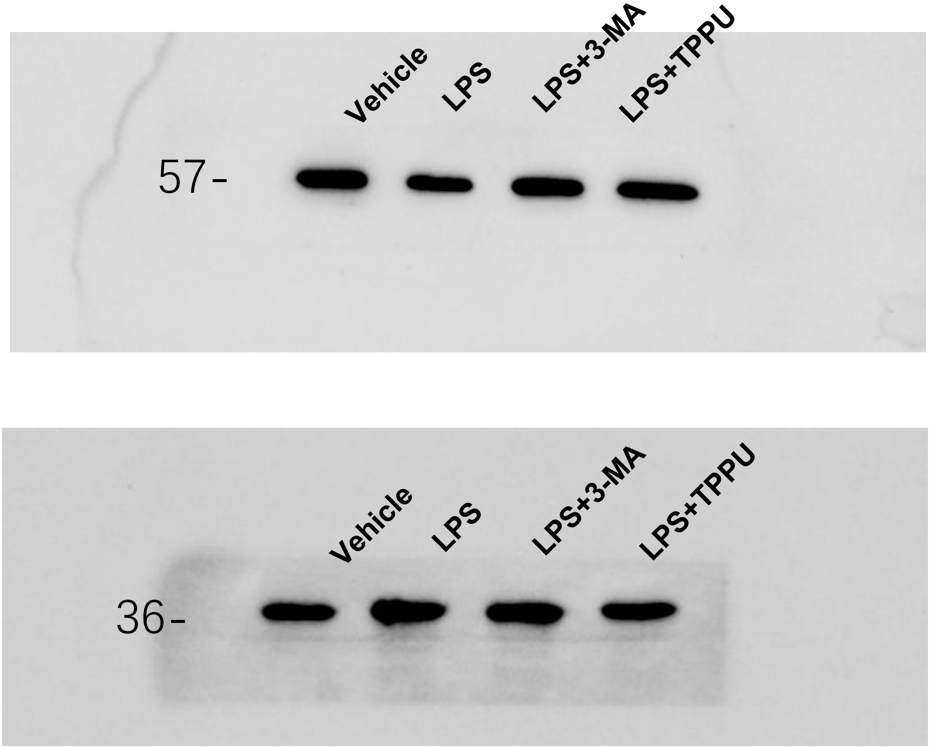

Supplementary Fig. 6 Unprocessed western blots and/or gels. Source Data for Supplementary Figure 2
